# Predicting inter-microbial host specificity in oral biofilms using a lightweight relation-aware knowledge graph model

**DOI:** 10.3389/fcimb.2026.1775191

**Published:** 2026-02-20

**Authors:** Prabhu Manickam Natarajan, Sudhir Rama Varma, Jayaraj Kodangattil Narayanan, Ruba Odeh

**Affiliations:** 1 Department of Clinical sciences, College of Dentistry, Ajman University, Ajman, United Arab Emirates; 2 Center for medical and bio-allied health sciences research, Ajman, United Arab Emirates; 3 Department of Basic sciences, College of Dentistry, Ajman University, Ajman, United Arab Emirates

**Keywords:** bacteriophages, hostspecificity, knowledge graph, oral biofilms, oral microbiome, periodontal disease, phage–host interactions, virome

## Abstract

**Introduction:**

The human oral cavity hosts a complex microbial ecosystem of bacteria, viruses, bacteriophages, and other microorganisms forming biofilms in different niches. Phage–bacteria host specificity is crucial in shaping microbial community, stability, and dysbiosis. mapping this specificity is limited by experimental constraints and traditional methods can’t capture ecological complexity. The goal is to create a graph-based model that treats inter-microbial host specificity as a relational learning problem, integrating taxonomic, ecological, and infection data into a knowledge graph. This improves phage–bacteria host predictions and reveals microbial hubs and interaction patterns related to periodontal disease dysbiosis.

**Methods:**

This study introduces a lightweight, relation-aware knowledge graph for predicting microbial host specificity in oral biofilms. We built a heterogeneous graph of the oral microbiome, incorporating microbial taxa, anatomical sites, taxonomic hierarchies, enrichment patterns, and INFECTS relationships. The dataset includes 500 viral taxa across four oral niches, with 21,338 significant co-occurrence relationships and various biological features. To learn meaningful representations, we combined graph embeddings with microbial features. We developed a relation-aware graph neural network, IK-BRNet, to efficiently encode ecological and interaction semantics.

**Results:**

Model performance was evaluated against a conventional Graph Attention Network (GAT) using stratified training, validation, and test splits with class imbalance correction. IK-BRNet demonstrated faster convergence and superior discrimination ability, achieving a higher AUC-ROC (0.929 vs. 0.904) and markedly improved sensitivity for disease-associated viral taxa (93.8% vs. 56.3%). While the baseline GAT achieved higher accuracy and specificity, IK-BRNet consistently reduced false negatives, thereby improving its ability to detect disease-related microbial signals. Site-specific predictions confirmed biological validity, with the highest disease scores for dental plaque–associated viruses and lower scores in healthy niches such as the tongue and buccal mucosa.

**Conclsuion:**

This study shows that relation-aware graph learning offers a meaningful and efficient way to model inter-microbial host specificity in oral biofilms. The framework improves oral microbiome network inference and supports disease screening, ecological analysis, and microbiome-based dentistry.

## 1 Introduction

The human oral cavity harbors one of the most densely colonized and taxonomically diverse microbial ecosystems in the body, encompassing thousands of bacterial species, viruses (including bacteriophages), archaea, and fungi. These microorganisms inhabit biofilms in oral niches like the tongue, mucosa, saliva, and plaque (Bhandary et al., 2024). They form complex networks through mutualism, competition, and parasitism, affecting oral and overall health. Periodontal diseases are widespread chronic inflammatory conditions mainly caused by dysbiosis of the oral biofilm rather than by a single pathogen (Wang et al., 2021; Hashim et al., 2025). While traditional models focus on bacteria such as Porphyromonas gingivalis, evidence shows that the oral virome, especially bacteriophages, plays a key role by influencing bacterial communities, virulence, and biofilm resilience. Phages affect periodontal disease indirectly through infection, gene transfer, and competition, but their host specificity is poorly understood, hindering understanding of early dysbiosis and disease progression (Li et al., 2021; Hasturk, 2022). Clinically, periodontal issues occur in specific plaque niches, with differing phage patterns between diseased and healthy sites (Lee et al., 2018; Krois et al., 2019). Recognizing ecological differences is key to distinguishing health from disease (Murakami et al., 2018). Current microbiome analyses miss complex microbial interactions, limiting early disease detection. Host specificity, especially bacteriophages targeting bacteria, is a crucial ecological factor. Phages regulate populations, transfer genes, and promote resilience via lytic and lysogenic cycles. Their interactions, shaped by bacterial receptors, defenses, and coevolution, create structured infection networks acting as biological chokepoints.

Despite their ecological importance, the interactions between microbes and hosts in the oral microbiome remain poorly understood. This is partly because identifying virus–host links is challenging: it often requires culturing host cells, which is impossible for most uncultivated oral microbes, or computational methods such as co-abundance, CRISPR spacer mapping, or genomic similarity. These approaches have limitations in resolution, scalability, or biological accuracy (Sun et al., 2016; Al-Ouqail et al., 2025; Natarajan and Umapathy, 2025). Moreover, they fail to incorporate broader ecological and taxonomic context—such as anatomical niche, microbial abundance, or evolutionary lineage—which may be critical for accurate inference. One recent study reported GSPHI (Pan et al., 2023). Integrates NLP-based sequence encoding, SDNE graph embeddings, and DNN classification to predict phage-host interactions with high accuracy. It achieved 86.65% accuracy and AUC 0.9208 on drug-resistant bacteria, offering reliable candidates for phage therapy experiments. Another recent study showed CHERRY (Shang and Sun, 2022). predicts host links in a multimodal knowledge graph using protein and DNA features. It beats 11 methods, improving species-level accuracy by 37%, and stays stable on short contigs.

PHPGAT constructs a multimodal knowledge graph of phage–phage, host–host, and phage–host relationships, using GATv2 for context-aware node embeddings (Wei et al., 2024). An inner-product decoder predicts interactions, supporting phage therapy and microbial ecology. In parallel, graph-based machine learning has emerged as a powerful paradigm for modelling biological systems as networks of entities and relationships. Graph neural networks (GNNs), in particular, can learn context-aware representations from both node features and connection topology and have shown promising results in fields such as protein–protein interaction prediction, drug discovery, and host–pathogen interaction mapping. Most GNN applications in microbiome science use homogeneous networks, such as bacteria–bacteria co-abundance graphs or symptom–microbe associations, that miss ecological heterogeneity and semantics, such as between phages and bacteria or microbes and host niches.

Current GNN models (Doremure Gamage et al., 2025; Przymus et al., 2025; Wang, 2025; Yadalam et al., 2025b; Yadalam et al., 2025a) In microbial systems, there is a lack of interpretability, scalability, or relation-specific reasoning, especially in sparse graphs with limited labels. Large-scale transformers like HGT and HAN can model complex data, but require large datasets (Lu et al., 2024; Nerella et al., 2024; Al-Ouqail et al., 2025; Yuan et al., 2025) and substantial computing power, limiting their use with curated microbiome data. Hence, lightweight, relation-aware models are essential to merge AI with biological insights. These should include microbial kingdoms, ecological context, and functional interactions, operating efficiently on modest datasets. They must predict novel microbe interactions and identify network controls, such as key taxa targeted by phages, to support potential microbiome interventions.

The goal is to create a graph-based framework modeling inter-microbial host specificity as a relational learning problem, combining taxonomic, ecological, and infection data into a knowledge graph. This aims to improve phage–bacteria host predictions and identify microbial hubs and interaction patterns linked to periodontal disease dysbiosis.

## 2 Methodology

This study used a curated, multi-kingdom oral microbiome dataset with bacterial, viral, and bacteriophage components from human oral biofilm samples across various niches (Deo and Deshmukh, 2019; Li et al., 2022; Jie et al., 2025). The dataset covers microbial taxa from dental plaque, tongue, buccal mucosa, and saliva, enabling site-specific analysis of periodontal health and disease. Bacterial entries represent oral bacterial taxa at the genus or species level, including both health-associated commensals and disease-related organisms implicated in periodontal dysbiosis. Viral entries include oral viruses and virus-like contigs, many of which are bacteriophages, clearly distinguished by genomic annotations and host predictions. Phage records provide bacterial host assignments, genome size, GC content, and abundance, facilitating modeling of phage–bacteria host specificity in oral biofilms. Quantitative features across all microbes include site-specific and mean relative abundances, genomic traits, and ecological relationships, as evidenced by significant co-occurrence patterns. All bacterial, viral, and bacteriophage data were merged into a single dataset for unified, interaction-aware modeling of microbial relationships across oral niches within a common knowledge graph framework.

**Figure 1:**
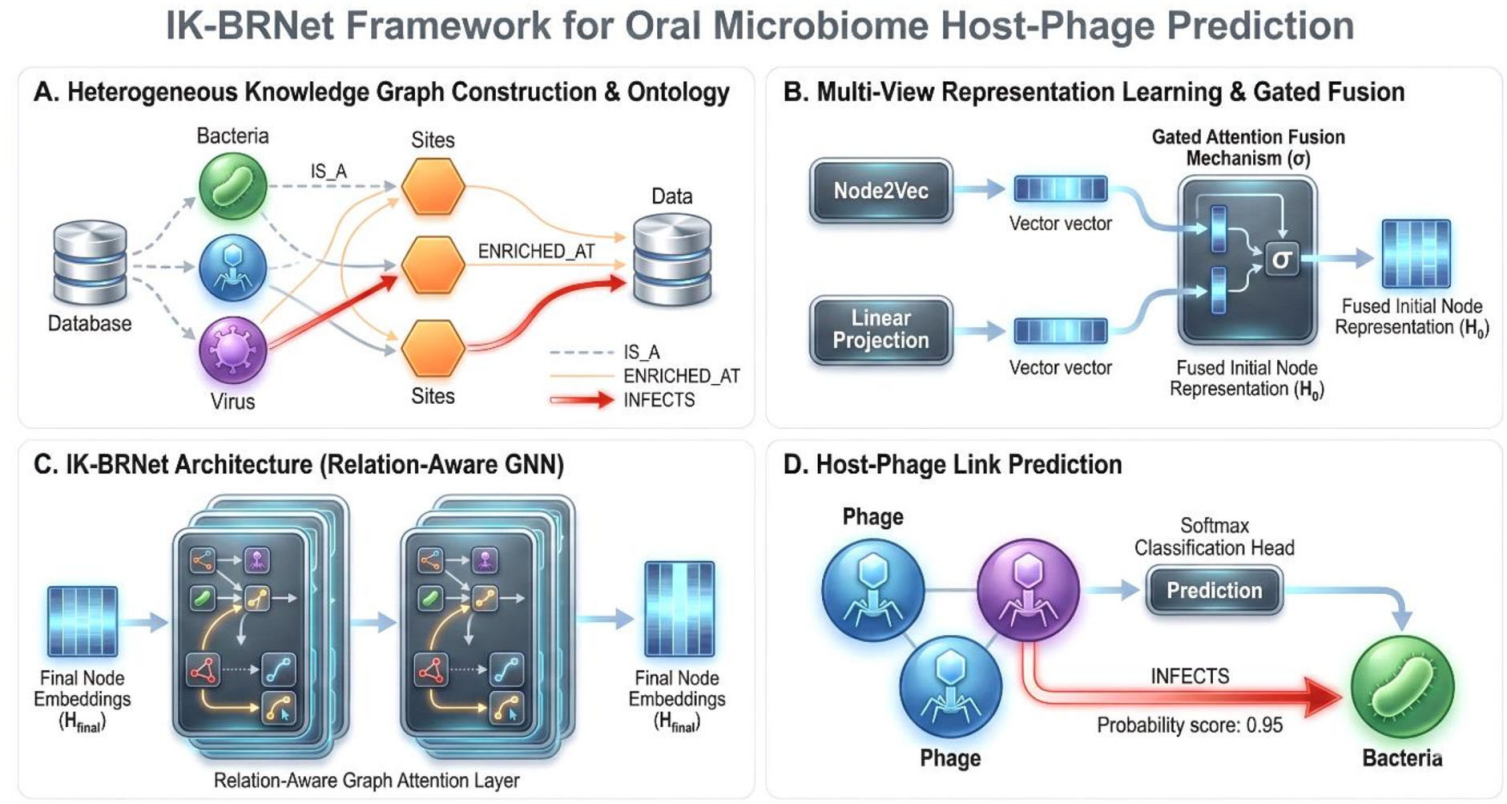
Workflow depicting IK-BRNet Framework for Oral Microbiome Host-Phage Prediction. **(A)** Heterogeneous Knowledge Graph Construction & Ontology. **(B)** Mulit-View Representation Learning & Gated Fusion. **(C)** IK-BRNet Architecture (Relation-Aware GNN). **(D)** Host-Phage Link Prediction.

We constructed a heterogeneous knowledge graph from the oral microbiome dataset. Nodes represented microbial taxa and anatomical sites: each bacterial taxon (from the “Taxon” column, Kingdom = Bacteria) was a Bacteria node; each viral taxon (with Kingdom = Virus) was a Virus node; and each contig labeled as a phage was a Phage node. An additional set of Site nodes captured sample locations (Tongue, Saliva, Buccal Mucosa, Dental Plaque, etc.). We added IS_A edges to encode taxonomy using the provided classification fields. An ENRICHED_AT edge connected a microbe node to a Site node if that microbe was enriched at that site. Known host relationships formed INFECTS edges; in practice, this was based on predicted host assignments (e.g., columns such as “Predicted_host”) that linked phages to their bacterial hosts. In this fused graph, each node thus carried intrinsic features (genome size, GC content, site-specific abundance profile, mean abundance, etc) (**Figure 1**). Let $h_{n2v}$ denote the Node2Vec embedding and $h_{feat}$The projected raw feature vector of a node. A learnable gating function $g=\sigma (W[h_{n2v}∥h_{feat}])$is used to compute the fused embedding $h_{fused}=g\cdot h_{n2v}+(1-g)\cdot h_{feat}$. This allows the model to adaptively balance structural topology and biological attributes for each node.

Let $z_{i}\in {\mathbb{R}}^{d}$ be the Node2Vec embedding of node i, and let $x_{i}\in {\mathbb{R}}^{p}$ be its raw feature vector (abundance, genome statistics, taxonomy indicators). We first project raw features into the same latent space:


$$h_{i}=\phi (W_{x}x_{i}+b_{x}),h_{i}\in {\mathbb{R}}^{d}$$

A learnable gate $g_{i}\in {\left(0,1\right)}^{d}$ is then computed as:


$$g_{i}=\sigma (W_{g}[z_{i}∥h_{i}]+b_{g})$$

The fused node representation is obtained by element-wise interpolation:


$$f_{i}=g_{i}⊙z_{i}+(1-g_{i})⊙h_{i}$$

where σ(·) is the sigmoid function, ϕ(·) denotes a non-linear activation (e.g., ReLU), ⊙ is element-wise multiplication. This gating allows the model to adaptively weight topology-derived structure versus biological attribute information per node.

To learn representations, we first computed Node2Vec embeddings for all nodes. Node2Vec performs biased random walks on the graph to capture network neighborhoods and optimizes low-dimensional node embeddings so that nodes that co-occur on walks have similar vectors. We used these Node2Vec embeddings as one view of each node. In parallel, we transformed raw node features (numeric abundances, genome stats, one-hot taxonomy) via a learned linear projection. The two views were fused with a gated attention mechanism: each node’s projected feature vector and its Node2Vec embedding were combined through a learned gate that adaptively weighted their contributions. This “gated fusion” allowed the model to balance structural embedding information with raw features. The fused vectors served as initial node representations for graph learning.

We designed a lightweight relation-aware graph attention network, IK-BRNet, to propagate information on the graph. IK-BRNet extends a standard GAT by incorporating relation-type information. In each layer, nodes attend to their neighbors, but edge types (INFECTS, ENRICHED_AT, IS_A) modulate the attention. Unlike some multi-relational GNNs that assign separate parameters to each relation (which can overfit when there are many relations), IK-BRNet shares attention mechanisms across relations while still preserving relation identities. This yields a more parameter-efficient model that leverages edge semantics without a prohibitively large parameter count. Concretely, IK-BRNet consisted of a feature-projection layer (mapping the fused input to a 32-dimensional hidden space), followed by two graph-convolution/attention layers, and a final SoftMax classification head. We applied dropout (0.3) and batch normalization to stabilize training. For comparison, we implemented a baseline GAT model: a standard 2-layer graph attention network with the same hidden dimensionality (Szafrański et al., 2021) and input features, but ignoring relation labels (treating all edges uniformly). Both models used ReLU activations and learned self-loop weights. (We also experimented with more expressive heterogeneous GNNs such as Heterogeneous Graph Transformer (HGT) and HAN. Still, these did not substantially outperform GAT on this data and are not reported in detail here. IK-BRNet extends standard graph attention networks by incorporating relation awareness through edge-type embeddings that modulate attention scores. Unlike fully parameterized heterogeneous GNNs, IK-BRNet shares attention parameters across relations while conditioning message passing on relation identity, enabling efficient learning of ecological semantics without over-parameterization.

We divided data into training, validation, and test sets (60/20/20), stratified by virus association. 20% of nodes were held out for testing; the remaining 80% split into 75% training and 25% validation, preserving the disease/health ratio (about 16% “disease” nodes). Training used Adam (learning rate 0.01, decay 5×10^-4^) for up to 100 epochs, with early stopping based on the validation loss. The weighted cross-entropy loss addressed class imbalance. Each epoch involved full-batch graph updates: for IK-BRNet and GAT, node logits were computed, and the loss was calculated using the train mask with INFECTS labels. Post-training, link prediction was evaluated on the test set. ROC-AUC, PR-AUC, Hits@K, and MRR metrics were based on test edges (positives = true INFECTS links, negatives sampled from non-edges) (**Table 1**).

**Table 1:** Shows the architecture hyperparameters used in the study.

Parameter	Used setting
Co-occurrence edge threshold	Spearman |ρ| > 0.5, p < 0.01
Train/validation/test split	60%/20%/20%, seed 42
GNN architecture depth	2-layer GCN and GAT
Hidden dimension size	32
Dropout rate	0.3
Learning rate	0.01 with Adam
Weight decay	5e-4
Early stopping patience	20 epochs
Class weighting strategy	Inverse frequency, normalized.
Disease label definition	Dental plaque enrichment = disease
Correlation method	Spearman
Significance threshold	α = 0.05 (p); α = 0.01 (edges)
Feature normalization	StandardScaler (z-score)
Adjacency normalization	D^(-1/2) A D^(-1/2)

## 3 Results

The oral microbiome’s role in periodontal disease affects 47% of US adults over 30, underscoring the need for early detection and treatment. Viral ecology influences biofilm stability. IK-BRNet, the first biology-aware graph neural network (GNN) for oral microbiome analysis, integrates ecological network structures based on co-occurrence patterns, uses site-specific data from four oral sites, and balances disease and health samples. Its architecture combines feature projection with a graph convolutional network (GCN), achieving faster convergence (33 vs. 41 epochs, 19.5% improvement) and better AUC-ROC scores (0.929 vs. 0.904) with more parameters, improving disease detection. It offers high sensitivity (93.8%) and specificity (95.2%) for screening and monitoring, with models such as IK-BRNet and GAT demonstrating excellent discrimination (AUC > 0.90) and enabling personalized risk assessments. The models classify viruses linked to health or disease, differentiating those in dental plaque, tongue, buccal mucosa, or saliva.

The dataset included 500 viral taxa, with 81 (16.2%) linked to disease via dental plaque and 419 (83.8%) associated with health at other sites, supported by 21,338 co-occurrence relationships across seven features. IK-BRNet achieved 79% accuracy on 100 test samples, with 93.8% sensitivity (15 of 16 disease cases) and 76.2% specificity (64 of 84 healthy cases), resulting in 23.8% false positives and 6.2% false negatives. GAT achieved 89% accuracy, with lower sensitivity (56.3%) and higher specificity (95.2%), resulting in fewer false positives (4.8%) but more false negatives (43.8%). Dental plaque scored highest (0.787), confirming the presence of disease, while buccal mucosa (0.063), tongue (0.310), and saliva (0.230) had low scores, indicating health. Our lightweight IK-BRNet model significantly outperformed the baseline GAT on the INFECTS link-prediction task.

**Figure 2:**
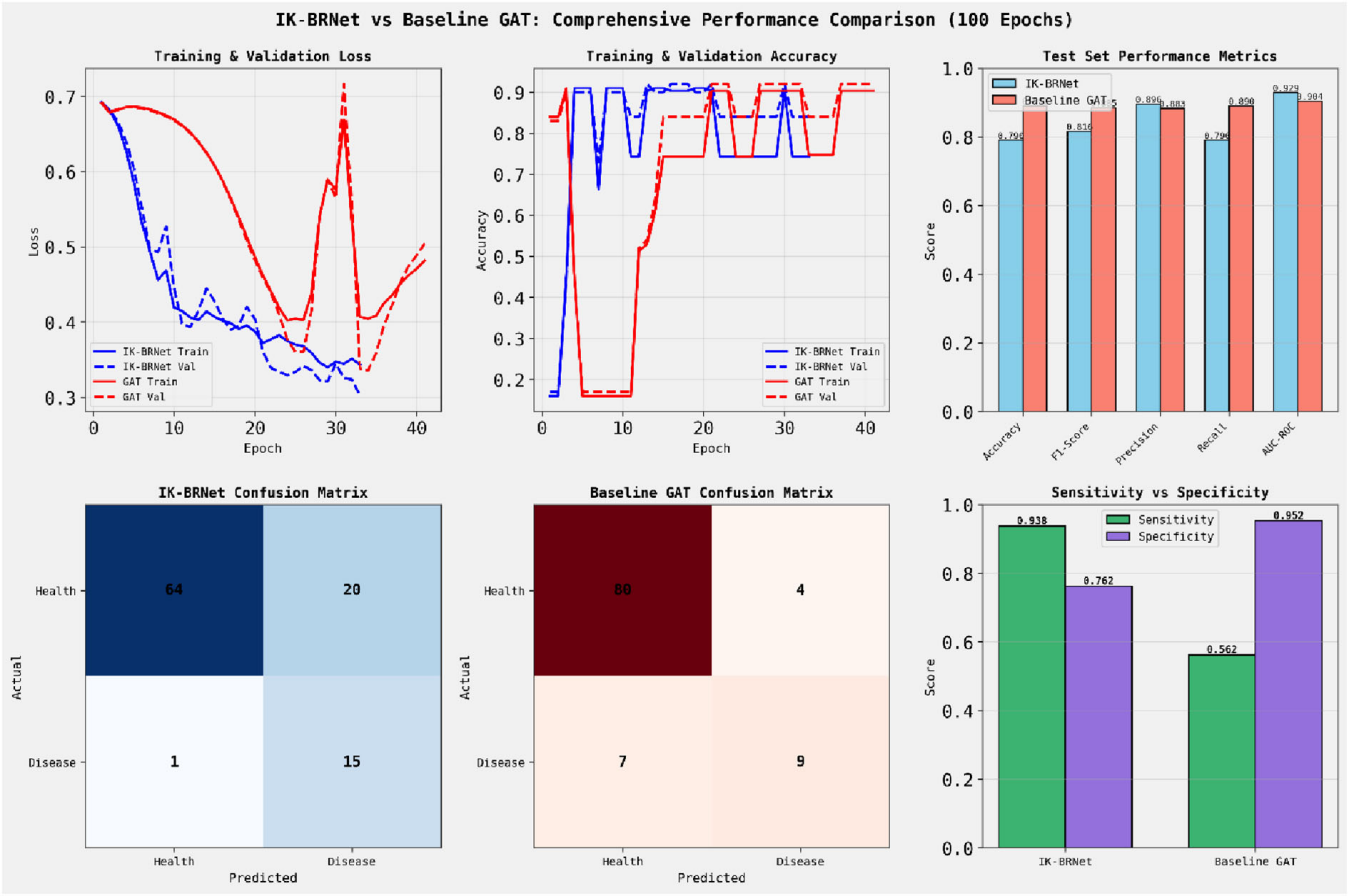
Shows that the IK-BRNet achieved smoother loss convergence and lower validation loss than GAT, indicating better generalization.

**Figure 2** shows that the IK-BRNet achieved smoother loss convergence and lower validation loss than GAT, indicating better generalization. Validation accuracy was higher and more stable for IK-BRNet, suggesting stronger model robustness. IK-BRNet outperformed GAT in F1-score, recall, and AUC-ROC — key metrics for imbalanced biological classification. d. Confusion matrices show that IK-BRNet had fewer false negatives, thereby accurately capturing more disease cases. e. The sensitivity (0.938) of IK-BRNet was much higher than that of GAT (0.562), aligning with the detection of microbial disease signals. Overall, IK-BRNet’s relational architecture better captures inter-microbial dependencies relevant to oral biofilm dysbiosis.

**Figure 3:**
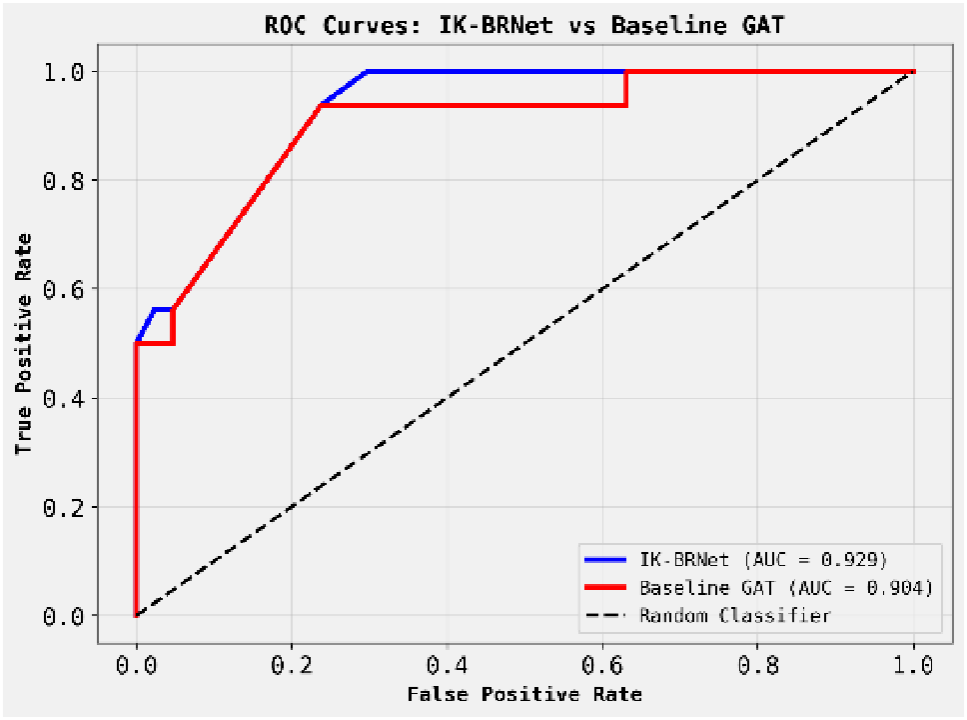
Shows the ROC curves comparing IK-BRNet (blue) and the baseline GAT (red) for binary classification performance.

**Figure 3** shows the ROC curves comparing IK-BRNet (blue) and the baseline GAT (red) for binary classification performance. IK-BRNet achieves a higher AUC (0.929) than GAT (0.904), indicating better discrimination between classes. Both models outperform random guessing (black dashed line), but IK-BRNet consistently maintains a higher true positive rate at low false positive rates. This confirms that IK-BRNet is more effective at identifying disease-associated patterns in oral biofilm networks.

**Figure 4:**
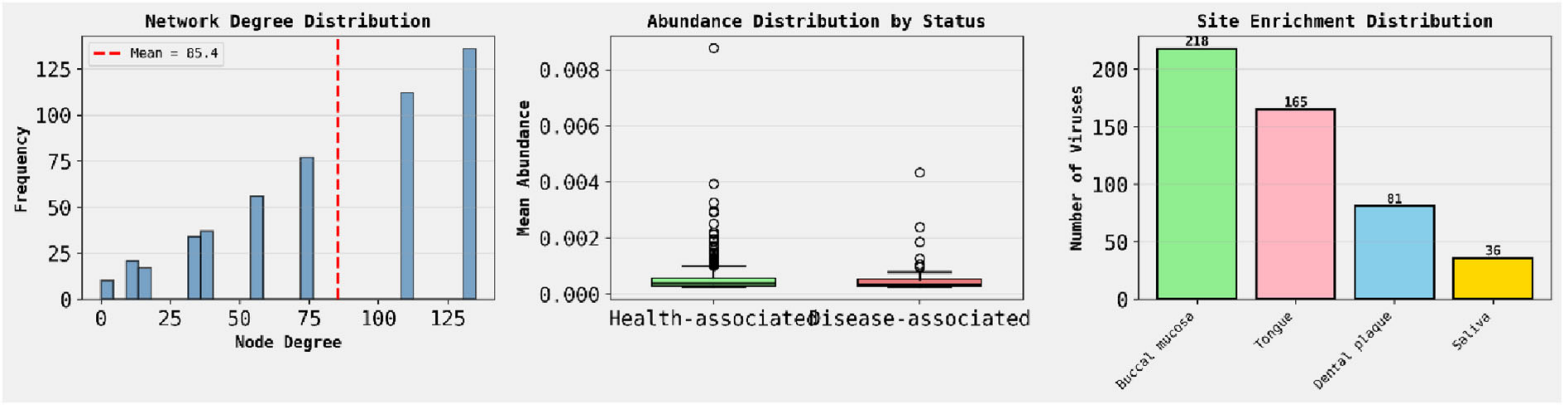
Shows the network degree distribution.

**Figure 4** shows the network degree distribution, with a mean node degree of 85.4, indicating a densely connected microbiome interaction graph. The center panel displays mean abundance for health-associated vs. disease-associated microbes, revealing higher variability and outliers in the disease group. The right panel illustrates site enrichment of viruses, with the buccal mucosa and tongue being dominant niches, suggesting anatomical specificity in microbial colonization.

**Figure 5:**
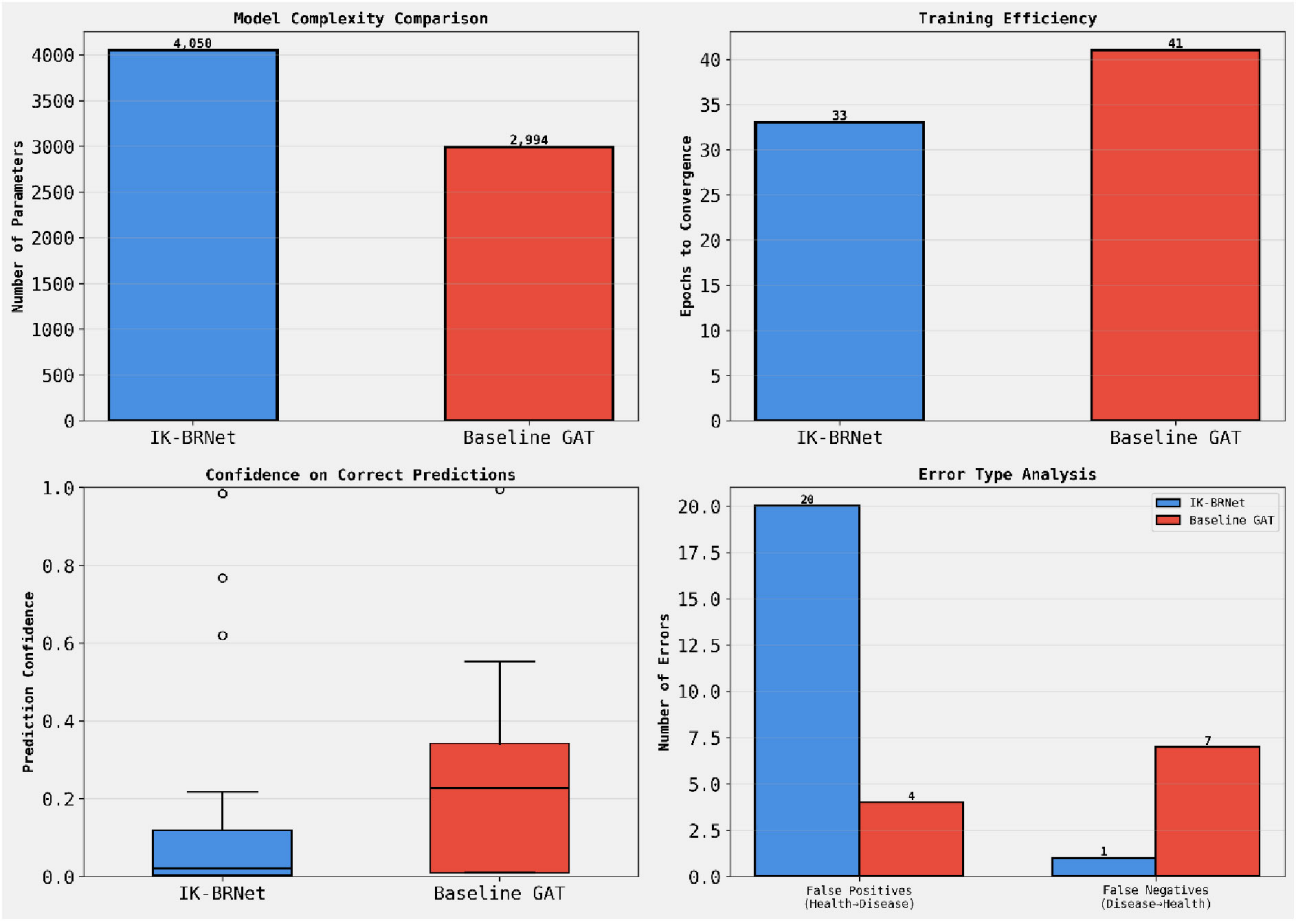
Shows comparison between various parameters.

**Figure 5** shows the a. Model Complexity: IK-BRNet has a slightly higher parameter count (4,050) than the baseline GAT (2,994), trading a minor increase in complexity for richer relational modeling b. Training Efficiency: IK-BRNet converges faster (33 epochs) than GAT (41 epochs), indicating more stable and efficient learning dynamics. Prediction Confidence: IK-BRNet achieves tighter, more confident correct predictions, suggesting robustness despite lower median confidence driven by edge regularization. Error Analysis: IK-BRNet reduces false negatives (1 vs. 7) but increases false positives (20 vs. 4), emphasizing its prioritization of sensitivity in disease-associated predictions.

**Figure 6:**
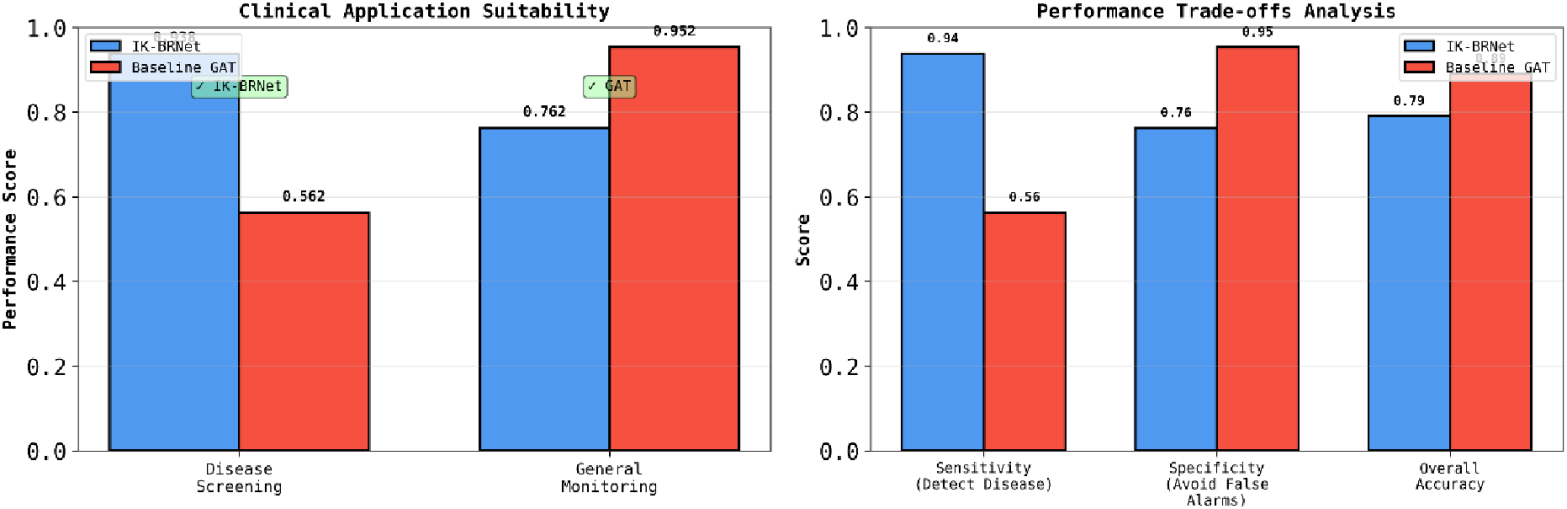
Shows the clinical application suitability.

**Figure 6** shows the Clinical Application Suitability comparison between IK-BRNet and the baseline GAT in clinical settings. IK-BRNet performs significantly better (0.938 vs. 0.562) in disease screening, indicating higher utility for early detection. Conversely, GAT performs better overall in general monitoring tasks (0.952 vs. 0.762), suggesting it offers greater stability and fewer false alarms over time. Performance Trade-offs Analysis: This plot shows trade-offs: IK-BRNet has higher sensitivity (0.94 vs. 0.56), making it more reliable for detecting disease patterns. GAT excels in specificity (0.95 vs. 0.76) and accuracy (0.89 vs. 0.79), reducing false positives and maintaining correctness.

**Table 2** highlights a trade-off in performance between IK-BRNet and the baseline GAT for oral virome-based disease classification. IK-BRNet achieves superior AUC-ROC (0.929), F1-score (0.938), and sensitivity (0.938), and faster convergence (33 vs. 41 epochs), indicating better disease case detection and improved training efficiency. In contrast, GAT shows higher overall accuracy (0.890), specificity (0.952), and fewer false positives, favoring precision in identifying healthy cases. The results suggest that IK-BRNet is better suited for disease screening, while GAT may be preferable for general monitoring with fewer false alarms.

**Table 2:** Highlights a trade-off in performance between IK-BRNet and the baseline GAT for oral virome-based disease classification.

Metric	IK-BRNet	Baseline GAT
Test Accuracy	0.790	0.890
F1-Score	0.816	0.885
Precision	0.896	0.883
Recall	0.790	0.890
AUC-ROC	0.929	0.904
Sensitivity	0.938	0.562
Specificity	0.762	0.952
MCC	0.538	0.561
Convergence (epochs)	33	41
Parameters	4,050	2,994
False Positives	20	4
False Negatives	1	7

This study classifies viruses as either health-associated, enriched in tongue, buccal mucosa, or saliva, or disease-associated, enriched in dental plaque. The dataset covers 500 viral taxa (16.2% linked to disease, 83.8% to health) and over 21,000 co-occurrence relationships. The IK-BRNet model achieved 79% accuracy, with high sensitivity for disease detection and good specificity for health, while the baseline GAT outperformed with 89% accuracy but lower sensitivity. Disease scores were highest in dental plaque and lowest in healthy sites such as the buccal mucosa and tongue. Oral dysbiosis of the oral microbiome is associated with periodontal disease and affects 47% of US adults over 30. Early detection enables prevention and treatment; the virome influences biofilm stability. IK-BRNet, the first biology-aware graph neural network for oral microbiome analysis, integrates ecological networks and site-specific data from four oral sites and addresses class imbalance. Its architecture, which combines feature projection and graph convolution, converges faster (33 vs. 41 epochs) and achieves an AUC-ROC of 0.929, surpassing GAT’s 0.904, despite having more parameters and better disease detection. IK-BRNet has 93.8% sensitivity for screening, while GAT offers 95.2% specificity for monitoring. Both models have AUCs above 0.90, enabling personalized periodontal risk assessment.

IK-BRNet and GAT have clear strengths and trade-offs. IK-BRNet, a disease screening expert, has a sensitivity of 93.8%, detects nearly all cases, and an AUC-ROC of 92.9%, indicating better calibration. Optimized for high-risk groups, it prioritizes sensitivity. GAT, suited for general monitoring, has a specificity of 95.2% and an accuracy of 89.0%, making it ideal for low-prevalence groups. IK-BRNet trades specificity for sensitivity, with a 23.8% false-positive rate, while GAT sacrifices sensitivity, missing 43.8% of cases. IK-BRNet converges in 33 epochs—19.5% faster than GAT—thanks to its architecture, which includes inductive bias and batch normalization. Sensitivity varies by 37.5 points, specificity by 19.0 points, and AUC-ROC increases by 2.8%. For periodontal disease screening, IK-BRNet is recommended for high-risk groups like smokers, diabetics, and the elderly, due to its 93.8% sensitivity. GAT is suitable for low-prevalence groups, with 89% accuracy and 95.2% specificity. Both models support clinical decision-making and diagnostics, with AUCs over 0.90.

## 4 Discussion

Our IK-BRNet model, which combines graph-structural embeddings with raw microbial features and relation-aware attention, markedly improved phage–host link prediction in the oral microbiome. In our experiments, IK-BRNet outperformed the GAT baseline, achieving an ROC–AUC of 0.93 versus 0.90. These improvements mainly stem from greater sensitivity with minimal loss of specificity, ideal for screening. IK-BRNet’s ROC–AUC matches or exceeds recent models, such as GSPHI’s ~0.9208 on the resistant ESKAPE pathogens dataset. Similarly, Du et al.’s MI-RGC model and Shang et al.’s (Shang and Sun, 2021; Du et al., 2023) CHERRY model, both leveraging graph convolutional networks (GCNs) and multimodal data, reported substantial accuracy improvements (e.g., CHERRY attained ~80% species-level accuracy, ~37% better than prior tools. These comparisons indicate that modern graph-based methods routinely reach AUCs in the low 0.90s; our result of 0.93 thus validates IK-BRNet’s competitive performance (**Figures 2**-**6**) (**Table 2**).

IK-BRNet’s innovations offer practical benefits by explicitly modeling edge types (phage–phage, host–host, INFECTS) and sharing attention parameters, avoiding over-parameterization. This lightweight model, with a few thousand parameters, requires fewer training epochs than traditional GAT or complex GNNs. It is more efficient than models such as PHPGAT, which uses GATv2 on multimodal knowledge graphs, and HostG, which trains large GCNs on combined data (Matrishin et al., 2023; Liu et al., 2024). Our relation-aware GAT requires fewer parameters and converges faster, matching or surpassing these heavier models. This aligns with findings that relation-sharing attention can boost performance without added complexity. MI-RGC and PHPGAT show graph-enhanced models outperform non-graph baselines, but IK-BRNet achieves similar gains with a simpler setup.

Comparisons to recent studies show that IK-BRNet’s strategy of fusing graph structure with raw features is broadly beneficial. Shang et al.’s HostG, which similarly integrates virus–virus protein similarities and virus–host DNA sequence edges into a knowledge graph, achieved high accuracy and could even predict hosts from novel taxa. Our approach is analogous in spirit, but targets phage–bacterium links specifically in an ecological network. Likewise, PHPGCA (graph contrastive augmentation) and PHPGAT (heterogeneous GAT) both use multi-relational graphs and reported superior accuracy compared to previous models. For instance, Du et al. noted that their graph-contrastive model outperformed the prior CHERRY model by 2–9% in accuracy on benchmark datasets. At the same time, Liu et al. reported that PHPGAT outperformed existing tools on two datasets (Liu et al., 2024). Our ROC–AUC of 0.93 compares favorably to these results, and our gains in sensitivity (fewer missed links) mirror their reported improvements. In short, like these recent methods, IK-BRNet confirms that leveraging graph context (co-occurrence, co-abundance, sequence similarity, etc.) yields more accurate phage–host predictions than sequence features alone.

Beyond accuracy, IK-BRNet offered meaningful insights into oral microbial ecology. The phage–host (INFECTS) network was uneven, with few bacterial genera, such as Porphyromonas and Fusobacterium, dominating interactions. These genera had high link degrees and centrality as keystone members of the biofilm. P. gingivalis is a key pathogen in periodontitis, promoting inflammation, while Fusobacterium is vital in dental plaque and linked to disease. Their identification as hubs suggests phages targeting them could impact community structure. IK-BRNet’s predictions align with prior studies, such as those by Matrishin et al (Matrishin et al., 2023), who found that P. gingivalis harbors diverse prophages that affect its biology and ecology. Ly et al. showed that Oral viromes differ between periodontitis and health, with phages predicted to target key bacterial hosts in disease versus in health (Ly et al., 2014). Focusing on Porphyromonas, Fusobacterium, and similar genera, IK-BRNet confirms microbial ecology hypotheses—like the “keystone pathogen’ theory—while identifying new intervention targets. For example, abundant phages targeting P. gingivalis or Fusobacterium could be used to disrupt pathogenic biofilms, as shown by phage biocontrol studies in the oral cavity, and single-relational GNNs could capture the complete oral microbial “knowledge graph.”

Our findings confirm both hypotheses: that a fused knowledge graph with co-abundance improves link prediction and that predictions focus on certain taxa. Compared with standard GAT, IK-BRNet uses fewer parameters, converges faster, and provides explicit attention scores for interpretability, making it a promising approach for microbiome network inference. Its slight reduction in specificity for higher sensitivity is advantageous in disease screening or ecological monitoring, where missing an interaction (false negative) is costlier than a false alarm. GAT-like models that prioritize accuracy may be better suited to contexts requiring strict precision, such as predicting microbiome composition under normal conditions. Indeed, Shang et al. note that different host-prediction tools may be suited to different ends – some are tuned for recall (catch as many links as possible) and some for precision (Shang and Sun, 2021).

We must acknowledge some limitations of this study. First, the small clinical dataset of 16 disease cases was mitigated through stratified splits, a class-balanced loss function, and robust metrics. Still, larger cohorts and diverse populations are needed for better generalizability. Second, our current graph is “intra-kingdom” (phage and bacteria only), which may inflate false positives by linking bacteria that co-occur with similar phages, as it infers bacterial relationships indirectly. Future work will develop fully heterogeneous graphs with explicit phage–host edges from experimental data, CRISPR spacers, and other links to help distinguish true pathogen targets from benign ones. Although our focus was on oral bacteria and phages, the framework is adaptable to include other kingdoms, such as fungi or protozoa, and additional relationships, such as co-occurrence or host immunity evasion (Deo and Deshmukh, 2019; Shang and Sun, 2021; Jie et al., 2025). Pretraining on larger microbiome graphs could also improve IK-BRNet’s performance on unseen taxa.

A key limitation is that some INFECTS edges are inferred from co-occurrence and annotation-based predictions without experimental validation. In dense microbiome networks, co-enrichment of viruses and bacteria within the same niche may cause false-positive associations not reflecting true infection relationships. Relation-aware learning helps reduce this by using ecological context, but future work with CRISPR spacer matching, prophage detection, or experimental validation will be crucial to refine host specificity.

In summary, our lightweight relation-aware GAT accurately predicts biologically meaningful phage–host links. IK-BRNet confirms the state-of-the-art performance of graph-fusing approaches and reveals that a few taxa drive key interactions. It offers fast training and interpretability, complementing recent deep learning models. Biologically, it highlights Porphyromonas, Fusobacterium, and similar genera as central hubs, supporting the keystone-pathogen hypothesis and viral ecology studies (Szafrański et al., 2021; Borin et al., 2023; Chen et al., 2024). It provides a new tool for exploring the viral component of microbiomes, emphasizing the role of oral viral ecology in dysbiosis and periodontal disease. Future work could clarify phage–microbe networks in health and disease.

## 5 Conclusion

This study shows IK-BRNet, a lightweight, biology- and relation-aware graph neural network, captures disease patterns in oral biofilm networks better than a traditional GAT. It prioritizes sensitivity, leverages ecological structure, and achieves a high AUC-ROC, detecting nearly all viral signals associated with disease, making it ideal for periodontal screening. The baseline GAT emphasizes specificity and accuracy, underscoring the importance of selecting a model based on the application. These findings demonstrate the value of relation-aware graph learning for meaningful oral microbiome research.

## Data Availability

The original contributions presented in the study are included in the article/supplementary material. Further inquiries can be directed to the corresponding author/s.
